# An acute crossover trial of passive movement training with and without blood flow restriction to determine the impact on postprandial glycaemia

**DOI:** 10.1113/EP093866

**Published:** 2026-08-19

**Authors:** Jay Railton‐Sowerby, Ewan Dean, Matthew Davitt, Jack Hayden, Ash Osborne, Donna Shrestha, David Cheneler, Christopher J. Gaffney, Paul Hendrickse

**Affiliations:** ^1^ Lancaster Medical School Lancaster University Lancaster UK; ^2^ East Lancashire Teaching Hospitals NHS Trust Blackburn UK; ^3^ Lancaster School of Engineering Lancaster University Lancaster UK

**Keywords:** BFR, blood flow restriction, exercise, glucose, glycaemic control

## Abstract

During glucose uptake into muscle, GLUT‐4 can translocate with stimulus from insulin or muscle contraction via exercise, but some exercise‐intolerant populations are unable to benefit from improved glycaemic control. We investigated how passive movement training (PMT) and passive movement training with blood flow restriction (PMT+BFR) could be used as an alternative method. The effects on blood lactate and insulin were also investigated. Eleven healthy males (26.9 ± 8.3 years, 25.7 ± 2.1 kg/m^2^) undertook a crossover trial of three 150‐min treatments, control (CTRL), PMT and PMT+BFR, on three separate study visits, separated by ≥24 h. Each participant arrived fasted and consumed a standardised high‐carbohydrate meal (522 kcal, 112.5 g carbohydrate). The PMT protocol involved 30 min of intermittent bilateral knee extension/flexion (1 min:1 min work/rest) at an angular velocity of 180°/s through an 80° range of motion. PMT+BFR used a pressure calibrated at 80% arterial occlusion. Blood glucose and lactate were measured every 5 min, with insulin at 0, 30 and 60 min. Acute changes in muscle thickness were recorded using ultrasound pre‐ and post‐intervention. There were no significant differences between the treatment groups for mean glucose (CTRL: 4.59 ± 0.48, PMT: 4.88 ± 0.82, PMT+BFR: 4.51 ± 0.90 mmol/L; *P* = 0.498). A statistically significant acute difference of muscle thickness (*P* = 0.00420) from pre‐ to post‐treatment was detected in PMT+BFR. While PMT did not improve postprandial glycaemia with or without BFR, the acute increase in muscle thickness after PMT+BFR suggests that it may be useful in other applications.

## Abstract (highlights)

- **What is the central question of this study?**
  What is the impact of passive movement training (PMT) and PMT combined with blood flow restriction (PMT+BFR) on postprandial glycaemic control?
- **What is the main finding and its importance?**
  PMT, even when paired with BFR, is insufficient to improve postprandial glycaemic control, suggesting that active muscle contraction or higher metabolic demand is essential for glucose disposal. However, the significant increase in muscle thickness observed with PMT+BFR identifies a potential therapeutic pathway for attenuating muscle atrophy in exercise‐intolerant populations.

## 1 INTRODUCTION

While control of blood glucose is typically considered for those with type 2 diabetes mellitus (T2DM), repeated large glucose excursions are linked to adverse health outcomes in those without T2DM, including endothelial dysfunction and increased oxidative stress (Avner & Robbins, 2025; Ceriello et al., 2008). It is thought that glucose toxicity from hyperglycaemia can impair insulin secretion, potentially contributing to the development of T2DM (Kawahito et al., 2009). Therefore, reducing postprandial glucose excursions is of critical importance in the prevention of, and perhaps treatment of, T2DM.

Blood glucose concentration is homeostatically regulated within a tight range (4–7 mmol/L in heathy populations when fasted) (Wrench et al., 2024). When glucose concentration increases beyond physiological requirements, insulin is released from the β‐cells of the pancreas. Action by insulin elicits a decrease in blood glucose by stimulating glucose uptake by skeletal muscle, the liver and adipose tissue (Rahman et al., 2021). Skeletal muscle is a major site of glucose disposal, accounting for up to 85% of glucose uptake (DeFronzo et al., 1981), and this occurs through GLUT‐4 translocation in response to increases in insulin (Richter & Hargreaves, 2013) or exercise (Wrench et al., 2024). More recent evidence also demonstrates that GLUT‐4 translocation can also be facilitated by a mechanical tension‐mediated mechanism present in muscle contraction and stretch which activates Rac1 GTPase (Sylow et al., 2015). Greater nitric oxide (NO) availability has been shown to mediate skeletal muscle glucose uptake via an insulin‐ and contraction‐independent pathway (Higaki et al., 2001). One factor which affects the delivery of glucose to facilitate uptake into muscle is blood flow (Baron et al., 1994; Sylow et al., 2017). Through increasing the mass action gradient and perfusion of the microvasculature via the Fick principle, increased blood flow reduces the diffusion distance of glucose to muscle fibres (Bonadonna et al., 1998).

Passive movement training (PMT), a form of physical activity whereby the individual undertaking the PMT does not voluntarily move their limbs, confers mechanical tension onto the muscle and induces a hyperaemic response (an increase in blood flow to an area of the body in physiological demand) to approximately 330% of resting values (Mortensen et al., 2012; Trinity & Richardson, 2019). Mechanical deformation of the limb in PMT causes the release of NO (Mortensen et al., 2012), causing an increase in blood flow which mimics (to a lesser extent) the increase in blood flow seen in active exercise (Trinity & Richardson, 2019). Therefore, we propose that passive movement will increase postprandial glucose uptake. Our rationale for this is that passive movement will improve the delivery of glucose via greater blood flow to the muscle, amplifying the insulin‐mediated glucose uptake response post‐meal. It is also possible that the mechanical deformation with movement of the limb will enable GLUT‐4 translocation via a stretch‐mediated mechanism using Rac1 GTPase. This contrasts with active exercise which stimulates the translocation of GLUT‐4 via an AMP‐activated protein kinase mediated mechanism (Wright et al., 2004).

Blood flow restriction (BFR) is most often used alongside exercise, but this method also induces a physiological response in the absence of active contraction; when a BFR cuff is removed, pronounced hyperaemia occurs (Wood et al., 1955), increasing sheer stress and potentiating NO‐mediated vasodilation. We hypothesise that this hyperaemic response should be greater compared to that seen with PMT alone, improving the delivery of glucose to the muscle membrane through greater microvascular perfusion and thus enhancing the post‐meal insulin‐driven uptake of glucose into muscle.

Studies with small sample sizes suggest that BFR in the absence of resistance training can mitigate muscle atrophy with immobilisation (Cerqueira et al., 2020), potentially through a cell swelling mediated mechanism (Bielitzki et al., 2021). While unproven in humans, it has been posited that muscle swelling may act as a hypertrophic stimulus through sensors detecting a cell membrane stretch (Schoenfeld, 2010; Schoenfeld & Contreras, 2014). Acutely, an increase in muscle thickness with limb occlusion should occur through a restriction of venous outflow; fluid is retained within the limb, adjusting the pressure gradient such that plasma moves from capillaries into the interstitial and intracellular spaces (Loenneke et al., 2012). Since muscle swelling correlates with long term hypertrophy outcomes from resistance training (Hirono et al., 2022), we included an exploratory measure of muscle thickness pre‐ and post‐intervention to see if PMT or PMT+BFR induced muscle swelling.

This study therefore aimed to examine the impact of PMT and PMT combined with BFR (PMT+BFR) on postprandial glycaemic control. To examine this effect, we conducted a crossover trial comparing PMT, PMT+BFR and a control (CTRL) whilst measuring venous blood glucose levels after a standardised meal. We examined the postprandial glycaemic responses by assessing metrics including area under curve, peak glucose concentration and time to peak glucose concentration. We observed effects on lactate, insulin and muscle thickness as secondary measures. It was hypothesised that (1) PMT would result in significantly lower postprandial glucose deviations from that at rest when compared with CTRL and (2) PMT+BFR would elicit a greater blunting effect on postprandial blood glucose than PMT alone.

To our knowledge this is the first study investigating the effect of non‐active knee extension and flexion on postprandial blood glucose. Evidence indicates that due to the involuntary effect of PMT, it may be pertinent for populations who cannot complete traditional voluntary exercise to perform PMT due to the potential for PMT to improve vascular function (Høier et al., 2010; Pedrinolla et al., 2022) and mitigate the skeletal muscle atrophy which occurs through inactivity (Barbalho et al., 2019). Hence, any additional benefit to postprandial glucose uptake may provide further rationale for this modality.

## 2 METHODS

### 2.1 Approval

Health screening was undertaken prior to enrolment into the study. Written informed consent was obtained during the screening process, and all study protocols were conducted within the boundaries of the 2024 revision of the *Declaration of Helsinki*. Ethical approval was granted by Lancaster University's Research Ethics Committee (registration FHM‐2024‐4434‐SA‐2). The trial was pre‐registered before the recruitment of the first participant on ClinicalTrials.gov (NCT06704126).

### 2.2 Subjects and inclusion criteria

An a priori power analysis indicated a minimum sample size of eight for a power of 0.8 at an α of 0.05 based on the estimated reduction of 2.0 (± 1.6) mmol/L post‐intervention using data from a previous meta‐analysis of passive stretching and blood glucose (Thomas et al., 2024).

Eleven healthy, male participants aged 18–40 years old were recruited to allow for an anticipated dropout rate of 27% (nearest whole number). For eligibility, subjects had to be medication‐free, non‐smokers and to have not donated blood recently. Participants’ phenotype data are presented in Table 1.

**TABLE 1:** Physiological phenotype of participants.

Parameter	Mean ± SD	Range (min–max)
Age (years)	26.9 ± 8.3	19–40
Mass (kg)	81.9 ± 9.0	68.0–93.9
Height (m)	1.79 ± 0.08	1.67–1.90
Fat (% of bodyweight)	16.1 ± 4.3	9.5–22.7
BMI (kg/m^2^)	25.7 ± 2.1	22.5–28.8
Muscle mass (kg)	65.2 ± 7.1	51.2–77.4
Muscle mass (% of bodyweight)	79.7 ± 4.1	73.5–86.1

### 2.3 Protocol

A single‐centre crossover trial was conducted. Treatments of CTRL, PMT and PMT+BFR were randomly ordered in allocation to participants. The PMT bout was used to observe the effects of PMT on postprandial glucose, while the PMT+BFR bout was used to observe if any additional benefit of BFR occurred. A CTRL bout was used for comparison. The study visits were on non‐consecutive days to enable participants to have normal activity levels and dietary intake in the day preceding testing. Time of testing was kept at the same time of day for each participant (±2 h) to account for circadian rhythm effects. Participants were instructed to arrive having fasted for at least 8 h to prevent interference from recently eaten meals on blood glucose readings. They were asked to refrain from ‘intense exercise’ and maintain normal levels of habitual physical activity for the 24 h prior to testing to ensure muscle glycogen did not differ at the start of each testing session. Each participant underwent all three treatments. The period of glucose measurement in each testing session was 150 min as performed by others looking at the effects of an intervention on postprandial glucose (Kong et al., 2019). The initial 30 min for each was identical. This consisted of a participant being fitted with an antegrade venous cannula into the vein in the cubital fossa by a suitably trained member of the research team, prior to consuming the standardised meal.

The nutritional composition of the meal was comprised of Kellogg's Frosties cereal (The Kellogg Company of Great Britain Ltd, Salford, UK; 120 g, 450 kcal: 104.4 g carbohydrates of which 44.4 g sugars; 5.5 g protein; 0.7 g fats; and 2.4 g fibre) and Alpro almond milk (Alpro (UK) Ltd, Kettering, UK; 300 mL, 72 kcal: 8.1 g carbohydrates of which 7.2 g sugars; 1.5 g protein; 3.3 g fats; and 0.9 g fibre). This meant that in total, the meal comprised 522 kcal: 112.5 g carbohydrate of which 51.6 g was sugars; 6.9 g protein; 4.0 g fats; and 3.3 g fibre. Based on the participants’ body mass and height, according to the Mifflin–St Jeor equation (Mifflin et al., 1990), participants should have a resting energy expenditure (REE) of 2170 kcal/day, and if sedentary a recommended daily intake of 2604 kcal (1.2 × REE). Thus, the meal accounts for 24% of their recommended intake if participants were sedentary.

Fasting blood samples were taken and blood sampling continued for the remaining 150 min at 5‐min intervals. Blood sampling was taken by drawing a 1 mL Luer‐lock syringe from the cannula and ejecting the syringe onto a non‐absorbent pad. A 20 µL sample obtained from the pad with a capillary tube and deposited into aliquots of haemolysing solution. Analysis via a benchtop glucose/lactate analyser was carried out (Biosen C‐Line GP+, EKF, Barleben, Germany). Additionally at 0, 30 and 60 min, a 3 mL vacutainer was taken for insulin analyses. The vacutainers were allowed to clot at ambient temperature for 15 min, then centrifuged for 10 min at 4°C, 1800 RCF (5702R Centrifuge, Eppendorf, Stevenage, UK). The cannula was flushed with 1 mL saline solution (0.9% sodium chloride) at 30‐min intervals. The residual saline solution was drawn from the cannula and disposed of prior to the next concurrent sample being taken to avoid sample dilution (Wrench et al., 2024).

### 2.4 Ultrasound muscle thickness

Ultrasound (Logiq 2, GE Healthcare, Wauwatosa, WI, USA) was used to measure muscle thickness prior to and immediately after the PMT/PMT+BFR/CTRL protocols. An L6‐12 linear array probe was used with a vascular preset and carotid sub‐setting. Adequate ultrasound conductive gel (Aquasonic 100, Parker Laboratories, Fairfield, NJ, USA) was applied topically allowing acoustic coupling with minimal skin compression. A pen mark was made to indicate the point at which the transducer was for the three readings to ensure consistency of probe placement for measurements both pre and post. The three sites used were arranged in a straight line perpendicular to the femur, on the participants’ left leg and approximately 10 cm proximal to the quadriceps tendon on visual inspection. The transducer was positioned at a 90° insonation angle. Once positioned with a satisfactory cross‐sectional image present, the freeze function was used alongside the measure function. The distance measured was from the femur boundary to the epimysium. Electromyography (EMG) data verified that the PMT protocol was passive and active contraction did not occur. An EMG sensor was attached to the skin above the vastus medialis in a convenience sample of participants (*n* = 4) and they were asked to perform a maximal leg extension contraction with no external load prior to the meal; then, 5 min of EMG data were taken during each of the protocols.

### 2.5 Passive movement therapy

An isokinetic dynamometer was used to perform the PMT protocol (S3, Biodex, Shirley, NY, USA). A custom fabricated attachment allowed PMT of both legs simultaneously. The PMT protocol parameters used were as per a previously published protocol (Burns et al., 2018), such that an 80° range of motion was used at an angular velocity of 180°/s (approximately 50 extension/flexions per minute). Research by Drouin et al. (2004) found that the Biodex S3 had excellent inter‐test and inter‐day reliability on all mechanical measures (intraclass correlation coefficient (ICC) ≥0.99) and excellent validity for all mechanical measures (ICC = 0.99). The PMT was used in bouts of 1 min, followed by 1 min of rest, for a total of 30 min to stimulate repeated hyperaemic responses. This repeated bout protocol has been demonstrated to induce repeated hyperaemic responses in response to PMT, in contrast to one transient seen at the beginning of continuous PMT (Burns et al., 2018).

### 2.6 Passive movement therapy ± BFR

The PMT aspect of the PMT+BFR treatment was identical to that of the PMT treatment. BFR cuffs were added (AirBands, VALD, Brisbane, Queensland, Australia). Research by Zhang et al. (2024) found that in 92 participants (male *n* = 46, female *n* = 46) the Vald AirBand cuffs had good validity (ICC = 0.85), excellent inter‐rater reliability (ICC = 0.97) and excellent test–retest reliability (ICC = 0.94). Participants self‐fitted the BFR cuffs as close to the inguinal fold as possible. The BFR cuffs were used to automatically measure arterial occlusion pressure and were calibrated to elicit 80% occlusion of arterial blood flow, the higher end of the range recommended by Patterson et al. (2019), to elicit the greatest hyperaemic response upon cuff deflation. The BFR was activated alongside the PMT in 5‐min blocks, followed by a 1‐min deflation. The BFR was used intermittently in this format, for the total 30 min of PMT.

### 2.7 Control

Participants sat stationary for the entire testing period. Any movement was kept to an absolute minimum.

### 2.8 Data analysis

Dependant variables measured were blood glucose, blood lactate, serum insulin and muscle thickness. An enzyme‐linked immunosorbent assay (ELISA) (Human Insulin ELISA Kit, CrystalChem, Elk Grove Village, IL, USA) was used to measure insulin. Samples were prepared and analysed with the manufacturer's protocol being followed. Absorbance was read at both 450 and 630 nm before subtracting the 630 nm absorbance readings from the 450 nm absorbance readings. The insulin concentrations are shown as micro‐units per millilitre (Dean et al., 2025). The area under the curve (AUC) was calculated for glucose and lactate using the trapezoidal method in GraphPad Prism 10.4.1 (GraphPad Software, Boston, MA, USA). The independent variables were the treatment conditions: CTRL, PMT and PMT+BFR with a secondary independent variable of time. Blood glucose and lactate measurements were obtained simultaneously via a benchtop blood analyser (Biosen C‐Line GP+, EKF, Barleben, Germany) from the same blood samples, at 5‐min intervals for the entire 150 min of testing. An additional measure of blood glucose over the time studied was obtained by calculation of the AUC for each participant undergoing each treatment. Calculation of AUC was performed for the full 150 min. Serum insulin measurements were taken at fasting (0 min), 30 min and 60 min. Muscle thickness was assessed before and after the relevant protocol. Shapiro–Wilk testing was undertaken to assess distribution of each dataset prior to analysis to determine normality. A repeated measures two‐way analysis of variance (ANOVA) was performed to evaluate differences in glucose concentrations and muscle thickness between treatments. Where significant interaction effects were identified, *post hoc* multiple comparison tests were conducted. Šidák *post hoc* analysis followed for any statistically significant results. Repeated measures one‐way ANOVA testing was performed on glucose area under curve, peak glucose, time to peak glucose, serum insulin, lactate area under curve, and peak lactate to examine if any differences between treatments were present. This was conducted using the GraphPad Prism AUC trapezoidal calculation function. Insulin concentration data were found to be non‐normally distributed, so Friedman's test was conducted using GraphPad Prism. All data are presented as means ± standard deviation.

## 3 RESULTS

### 3.1 Glucose

**FIGURE 1:**
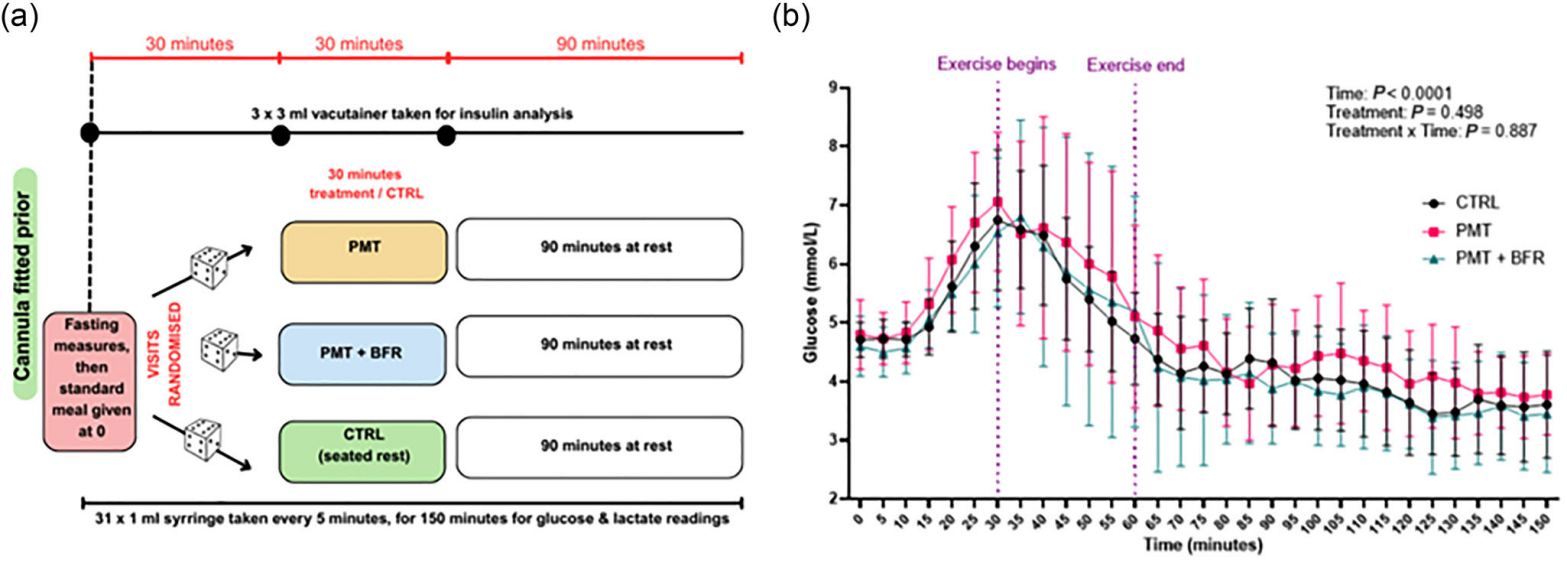
(a) A schematic illustration of the study design. (b) Mean glucose concentrations across the three treatment groups with a standardised meal consumed from time point zero minutes. Time point 30 min is where PMT/PMT+BFR protocols commenced in the relevant treatment groups. CTRL, control; PMT, passive movement training; PMT+BFR, passive movement training with blood flow restriction.

Baseline blood glucose concentrations were similar (Figure 1) across all three treatment groups (CTRL, 4.59 ± 0.48 mmol/L; PMT, 4.88 ± 0.82 mmol/L; PMT+BFR, 4.51 ± 0.90 mmol/L) (*P* = 0.490). Two‐way ANOVA tests of mean glucose concentrations per participant, per treatment also showed no significant time × treatment interaction (*P* = 0.90) or treatment interaction (*P* = 0.498). A significant time effect was detected (*P* < 0.0001).

**FIGURE 2:**
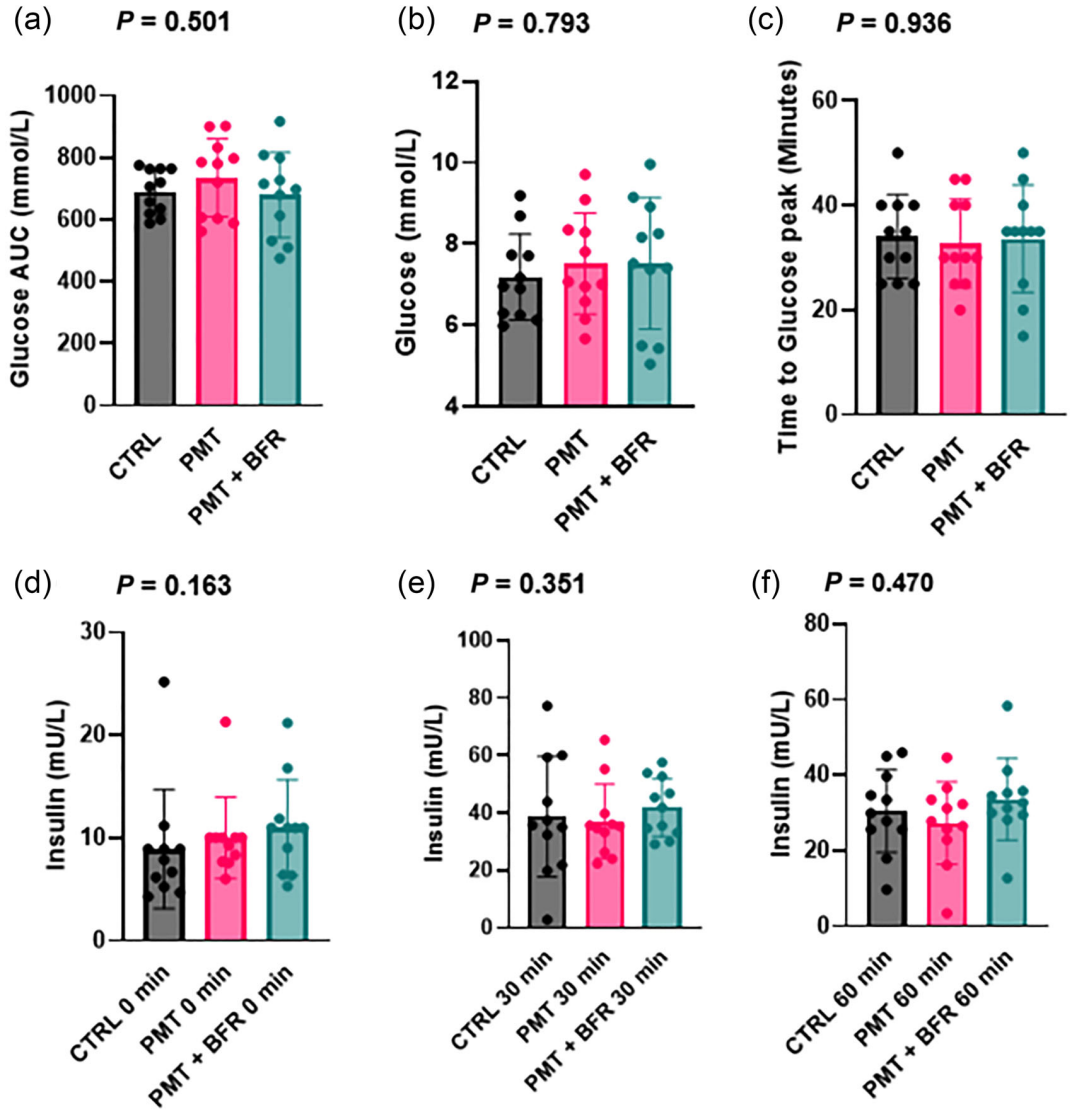
(a) Area under curve of glucose data, representative of the entire 150 min for which samples were taken. (b) Peak glucose concentrations, calculated as at the time point of individual participant peak glucose concentration. (c) Time to peak glucose concentration. (d–f) Insulin concentrations at 0 min (fasted) (d), at the commencement of exercise (30 min) (e), and end of exercise (60 min) (f). CTRL, control; PMT, passive movement training; PMT+BFR, passive movement training with blood flow restriction.

No significant difference was observed via a one‐way ANOVA (*P* = 0.501) as shown in Figure 2a. Peak glucose concentration was also compared via a one‐way ANOVA, with no statistical difference detected between the three treatment groups (CTRL 7.18 ± 1.06 mmol/L; PMT 7.52 ± 1.25 mmol/L; PMT+BFR 7.52 ± 1.62 mmol/L; *P* = 0.793) as displayed in Figure 2b. No significant differences were detected for time taken to reach peak glucose concentration via one way ANOVA analysis (CTRL 34.09 ± 8.01 min; PMT 32.73 ± 8.47 min; PMT+BFR 33.64 ± 10.27 min; *P* = 0.936) as shown in Figure 2c.

Normality testing determined that insulin measurements were non‐normally distributed. Friedman's test was carried out to assess for differences between the treatments. No significant differences were detected for any time points (0 min, *P* = 0.163; 30 min, *P* = 0.351; 60 min, *P* = 0.470) as shown in Figure 2d, e and f, respectively.

### 3.2 Lactate

**FIGURE 3:**
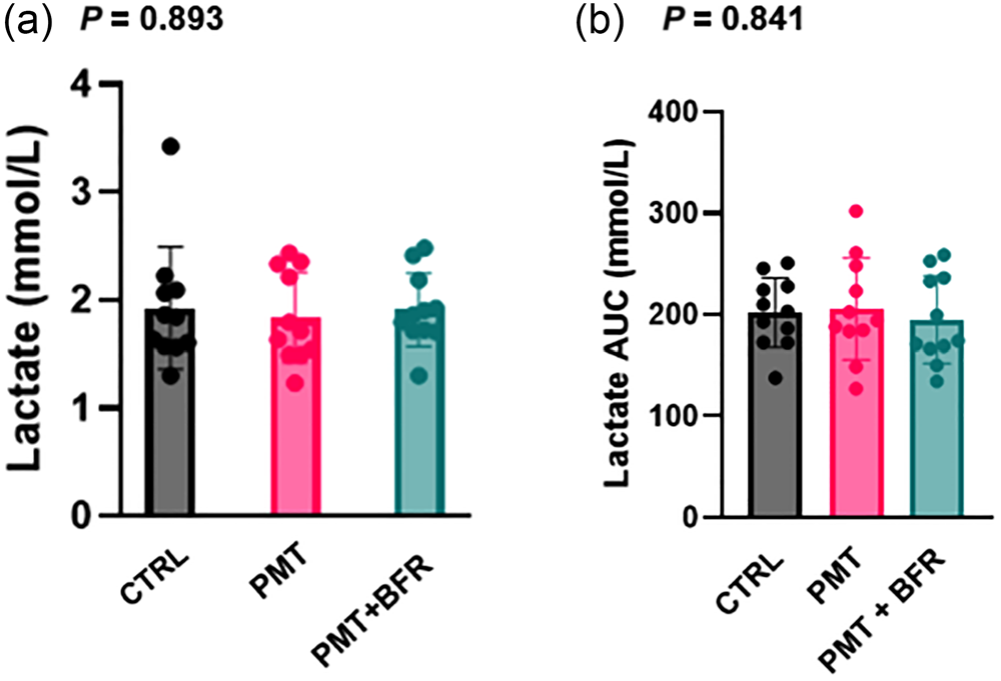
(a) Peak lactate concentrations across three treatment groups. (b) Area under curve of lactate data, representative of the entire 150 min for which samples were taken. CTRL, control; PMT, passive movement training; PMT+BFR, passive movement training with blood flow restriction.

Peak lactate concentrations were examined for differences across the three treatment groups via one‐way ANOVA. No statistically significant differences were detected (*P* = 0.893) as shown in Figure 3a. In the interest of a comprehensive examination of lactate concentrations throughout the protocols, area under curve analyses were also conducted. Calculations were carried out for the full 150 min of the protocol. No significant difference was observed between the three treatment groups (*P* = 0.841) as shown in Figure 3b.

### 3.3 Muscle thickness

**FIGURE 4:**
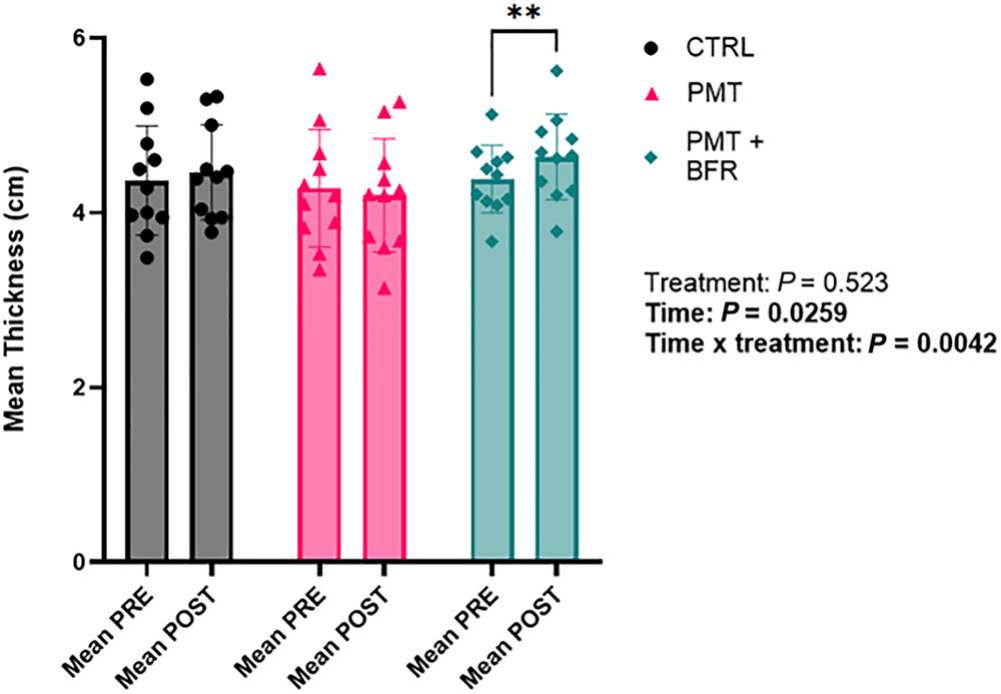
Muscle thickness changed over time between treatments. Undertaking PMT+BFR displayed significant acute increases from pre‐ to post‐protocol in muscle thickness (*P* = 0.00420). **P < 0.01. PMT, passive movement training; PMT+BFR, passive movement training with blood flow restriction; CTRL, control.

A two‐way ANOVA for muscle thickness revealed a statistically significant time × treatment interaction (*P* = 0.0042). *Post hoc* analyses revealed a difference in muscle thickness between mean Pre and mean Post in the PMT+BFR treatment (*P* = 0.0016). Cohen's *d* was used to evaluate effect size of pre to post muscle swelling in PMT+BFR and was calculated as 1.05 (Figure 4).

## 4 DISCUSSION

Findings showed no significant differences in the rate of glucose disposal across treatment groups as measured by area under curve, peak glucose and average glucose. These results did not align with the initial hypotheses which predicted (1) undertaking PMT would elicit a blunted postprandial glucose response and (2) that this effect would be exaggerated further with the addition of BFR. However, the present study determined that performing PMT alongside BFR resulted in a significant increase in muscle thickness (*P* = 0.0042). These results demonstrate that neither PMT nor PMT+BFR in this form were effective in amplifying glucose uptake into muscle, but PMT+BFR does induce muscle swelling acutely.

### 4.1 Glucose disposal and possible mechanistic explanations

A lack of difference in blood glucose between visits suggests that movement may involve active muscle contraction to elicit a greater rate of glucose disposal, as is shown with conventional exercise (Frank et al., 2021; Richter & Hargreaves, 2013).

The rationale for undertaking this study was multifaceted. Previously PMT has been shown to increase blood flow to approximately 330% of that at rest (a hyperaemic effect) (Trinity & Richardson, 2019). The hyperaemic effect observed from PMT is thought to be caused by NO, because inhibition of NO has been shown to reduce the hyperaemic response to PMT by approximately 90% (Mortensen et al., 2012). We attempted to investigate if using PMT to increase blood flow from resting levels postprandially would elicit an increased rate of glucose disposal. However, it should be noted that although blood flow is increased to approximately three times that seen at rest with PMT, conventional exercise increases blood flow to approximately 17‐fold, likely to satisfy oxygen and substrate delivery (Boushel et al., 2000; Mortensen et al., 2012). It may be that this elevation in blood flow with PMT is insufficient to stimulate a meaningful reduction in blood glucose via uptake into skeletal muscle.

Another potential explanatory mechanism is the lack of metabolic substrate requirement in skeletal muscle with PMT, compared to active contraction. Active contraction requires cross‐bridges (from myosin heads to actin binding sites) to be formed and broken to facilitate contraction (Lemaire et al., 2019). This active contraction is an energy intensive process due to required active calcium ion transport. With PMT, it is likely that any metabolic cost is significantly less than that of active contraction. This is due to the mechanical work and kinetic energy being delivered by external force, rather than the muscle itself. Due to the contraction being passive and not active in the present study, it is likely that glucose disposal rate was not increased because no active contraction occurred and thus there was no elevation in demand to replace glycogen stores (Richter & Hargreaves, 2013).

Another aspect of PMT which may explain why glucose disposal was not greater compared to CTRL is an insufficient stretch used in the PMT protocol. As per a previous published protocol, this study utilised a range of motion for leg flexion/extension of 80° (Burns et al., 2018). This range of motion ensured all participants could achieve the full range without excessive discomfort when manipulated through the range by a force exerted by the isokinetic dynamometer, and this has been found to induce an increase in blood flow. A meta‐analysis from 2024 showed an 8.85% reduction in acute glucose measures with passive stretching groups relative to controls (Thomas et al., 2024). Mechanistically, stretching may play a role in the reduction in acute glucose metrics by a sequence of events whereby stretching exercises would increase Ca^2+^ release from vascular endothelium, leading to NO release (Hotta & Muller‐Delp, 2022). However, had a larger range of motion been used to elicit a more exaggerated stretch, this could have stimulated further NO release and subsequent glucose uptake (Thomas et al., 2024).

A separate but similar mechanism which may be pertinent to consider given this study's BFR aspect, is the haemodynamic fluctuations seen in stretching. It has previously been observed that in the stretch phase a decrease in blood flow occurs and therefore relative ischaemia, which decreases glucose uptake. However, upon release of the stretch an influx of blood (hyperaemia) occurs and therefore an increase in overall glucose uptake (Pellinger & Emhoff, 2022). This suggests the use of BFR could increase glucose uptake overall due to the ischaemia of BFR application and reactive hyperaemia following BFR release. Reactive hyperaemia could then cause an increase in overall glucose uptake. As with PMT alone, however, it seems that these elevations in blood flow alone are insufficient to drive further glucose uptake into skeletal muscle.

### 4.2 Lactate

No significant differences in lactate were observed between treatments. This contrasts with previous findings of BFR with resistance exercise which show a positive relationship between BFR application time and blood lactate (Neto et al., 2017). While total limb occlusion leads to elevated lactate through ischaemia, only 80% arterial restriction was used in this protocol, and thus it is likely that active muscle contraction during exercise is necessary for the elevation in lactate with BFR to achieve full arterial restriction.

### 4.3 Insulin responses

A key aspect of this study was to examine insulin responses in synergy with glucose responses. No significant differences were detected in insulin responses across treatments. It was hypothesised that, particularly when combined with BFR, passive movement would improve postprandial glucose disposal and would cause a different insulin response from that of the control. The measurement of glucose alongside insulin was important to investigate to see whether any observed improvements in glycaemic control were mediated by changes in pancreatic insulin secretion or through movement‐mediated glucose uptake alone. This method allowed for the assessment of the role that endocrine regulation played in uptake of glucose compared to any peripheral and mechanical mechanisms.

### 4.4 Muscle thickness

In comparison to CTRL and PMT without BFR, it was found that BFR application elicited a significant increase (*P* = 0.0042) in acute muscle swelling from pre‐ to post‐PMT protocol. Several mechanisms could contribute to the acute swelling observed, one being that by applying BFR, the venous outflow from the limb is completely occluded; however, only 80% arterial occlusion is applied. The net result is that there is still blood flow to the limb and therefore the quadriceps and so oedema may occur. The osmotic influx would then result in cellular swelling and acute muscle swelling. Previous literature has shown similar acute effects when BFR was combined with active exercise (Cleary et al., 2022). The acute muscle swelling findings observed may suggest potential therapeutic applications of BFR; however, further research is required.

Interestingly, a potential mechanism for how BFR elicits a hypertrophic/atrophy attenuating effect is linked with cell swelling. Previous findings have shown load‐matched BFR exercise caused significantly more acute muscle swelling than exercise without BFR (Haddock et al., 2020). Cell swelling has been proposed as an independent stimulus for muscle growth (Schoenfeld, 2010; Schoenfeld & Contreras, 2014); however, any potential mechanism between cell swelling and hypertrophy is yet to be demonstrated (Loenneke et al., 2012). Previous studies provide inconsistent findings regarding the use of BFR in bed rest for the purpose of mitigating atrophy (Barbalho et al., 2019; Fuchs et al., 2025). Notably, Barbalho et al. (2019) implemented PMT+BFR and found a blunting of atrophy, whereas Fuchs et al. (2025) used BFR alone and saw no impact with this intervention. This difference warrants further research to elucidate the effectiveness of PMT+BFR for mitigation of muscular atrophy.

### 4.5 Limitations

Confirmation of a definitive hyperaemic response is only possible via Doppler ultrasound arterial pulse speed measurement (Gifford & Richardson, 2017). Due to the size, shape and location of the BFR cuffs, femoral arterial velocity measurements could not be obtained due to turbulent blood flow in proximity to the bifurcation, and therefore this led to inaccurate Doppler readings. However, the hyperaemic response to PMT is a well‐established physiological phenomenon (Burns et al., 2018; Gifford & Richardson, 2017; Høier et al., 2010; Mortensen et al., 2012; Trinity & Richardson, 2019) to the extent that it is used to test peripheral vascular function (Gifford & Richardson, 2017). Hence it is assumed that the same response occurred in this investigation. The effects of BFR on glucose uptake and muscle thickness were not measured in the absence of PMT, and we therefore cannot say that the inclusion of PMT with BFR induces greater muscle swelling than BFR alone. Finally, the indirect measurement of glucose uptake into muscle is a methodological limitation. Blood glucose was measured but this was only a proxy for muscle glucose uptake, instead of direct measurement with radioactive tracers and muscle biopsies. However, the only differences between groups were the movement and occlusion of the lower limbs, and therefore any possible differences in blood glucose were likely due to uptake into muscle. Lastly, it was not possible to blind the participant to the treatment being delivered simply due to haptic feedback from limb movement and BFR cuff application.

### 4.6 Conclusion

In summary, the findings of this study suggest that passive exercise, with or without intermittent occlusion, is not an effective method for reducing postprandial glucose excursions, whilst also highlighting the significant acute muscle swelling effect of BFR. These findings indicate that the use of exercise to reduce the postprandial glucose rise must include active contraction, greater increases in blood flow or an exaggerated muscle stretch.

## AUTHOR CONTRIBUTIONS

Conceptualisation: Paul Hendrickse and Christopher J. Gaffney. Methodology: Paul Hendrickse, Christopher J. Gaffney and David Cheneler. Validation: Ewan Dean. Formal analysis: Jay Railton‐Sowerby, Ewan Dean and Matthew Davitt. Investigation: Jay Railton‐Sowerby, Ash Osborne, Jack Hayden and Donna Shrestha. Resources: Paul Hendrickse and David Cheneler. Writing‐Original Draft: Jay Railton‐Sowerby and Matthew Davitt. Writing‐Review and Editing: Jay Railton‐Sowerby, Ewan Dean, Matthew Davitt, Jack Hayden, Ash Osborne, Donna Shrestha, David Cheneler, Christopher J. Gaffney and Paul Hendrickse. Visualisation: Matthew Davitt. Supervision: Paul Hendrickse and Christopher J. Gaffney. Project administration: Paul Hendrickse and Christopher J. Gaffney. All authors have read and approved the final version of this manuscript and agree to be accountable for all aspects of the work in ensuring that questions related to the accuracy or integrity of any part of the work are appropriately investigated and resolved. All persons designated as authors qualify for authorship, and all those who qualify for authorship are listed.

## CONFLICT OF INTEREST

None declared.

## FUNDING INFORMATION

None.

## GENERATIVE AI STATEMENT

We confirm that generative AI was not used in preparing this article.

## Data Availability

All original data reported in this manuscript are available from the corresponding author upon reasonable request.
